# Distinct Clinical Significance of Minimal Residual Disease Detected by 7NB-mRNAs Expression in Bone Marrow at Different Time Points of High-Risk Neuroblastoma Patients

**DOI:** 10.3390/biology15050427

**Published:** 2026-03-05

**Authors:** Cho Yee Mon, Kaung Htet Nay Win, Akihiro Nishimura, Naoko Nakatani, Akihiro Tamura, Nobuyuki Yamamoto, Nanako Nino, Suguru Uemura, Atsuro Saito, Toshiaki Ishida, Takeshi Mori, Daiichiro Hasegawa, Keisuke Okuno, Yoshiyuki Kosaka, Kikyo Ishizawa, Mayuno Umemoto, Taisei Matsui, Ayaka Nagatani, Noriyuki Nishimura

**Affiliations:** 1 Department of Public Health, Kobe University Graduate School of Health Science, Kobe 654-0142, Japan; choyeemon91@gmail.com (C.Y.M.); kaunghtetnaywin.tetsuya@gmail.com (K.H.N.W.); k.ishizawa02@gmail.com (K.I.); ut4326@gmail.com (M.U.); tunma141204mga@gmail.com (T.M.); 0187nyaga.yfa@gmail.com (A.N.); 2 Department of Pediatrics, Kobe University Graduate School of Medicine, Kobe 650-0017, Japan; ak2213@med.kobe-u.ac.jp (A.N.); nknaka@med.kobe-u.ac.jp (N.N.); atamura@med.kobe-u.ac.jp (A.T.); nyama@med.kobe-u.ac.jp (N.Y.); 3 Department of Hematology and Oncology, Kobe Children’s Hospital, Kobe 650-0047, Japan; nknino_kch@hp.pref.hyogo.jp (N.N.); sguemura_kch@hp.pref.hyogo.jp (S.U.); saitou_kch@hp.pref.hyogo.jp (A.S.); ishida_kch@hp.pref.hyogo.jp (T.I.); tsmori_kch@hp.pref.hyogo.jp (T.M.); hasegawa_kch@hp.pref.hyogo.jp (D.H.); 4 Division of Pediatrics and Perinatology, Tottori University Faculty of Medicine, Yonago 683-8504, Japan; kokuno53@tottori-u.ac.jp; 5 Faculty of Rehabilitation, Kobe Gakuin University, Kobe 651-2180, Japan; kosaka_kch@hp.pref.hyogo.jp

**Keywords:** neuroblastoma (NB), minimal residual disease (MRD), bone marrow (BM), neuroblastoma-associated mRNAs (NB-mRNAs), droplet digital PCR (ddPCR), end of high-dose chemotherapy (EOH), end of consolidation (EOC)

## Abstract

Patients with high-risk neuroblastoma (NB) continue to have long-term survival rates below 60%, with relapse occurring in more than half of patients, likely driven by chemoresistant minimal residual disease in the bone marrow (BM-MRD). Although several quantitative PCR (qPCR) and droplet digital PCR (ddPCR) assays measuring different but overlapping sets of NB-associated mRNAs (NB-mRNAs) have shown a significant prognostic value at various time points, the optimal combination of MRD markers (a set of NB-mRNAs) and evaluation timing remains unclear. In the present study, 89 bone marrow samples were collected from mostly overlapping 30 high-risk NB patients at four time points: diagnosis (Dx), end of induction (EOI), end of high-dose chemotherapy (EOH), and end of consolidation (EOC). BM-MRD was assessed with a 7NB-mRNAs ddPCR assay quantifying CRMP1, DBH, DDC, GAP43, ISL1, PHOX2B, and TH mRNAs. BM-MRD at EOH and EOC time points was significantly associated with relapse. Moreover, patients with higher BM-MRD levels at EOH (7NB-mRNAs ≥ 3.5) and EOC (7NB-mRNAs ≥ 3.5) time points had significantly inferior 3-year event-free survival (EOH, *p* = 0.003; EOC, *p* = 0.033). These results indicate that EOH and EOC are clinically informative evaluation time points for BM-MRD detected by 7NB-mRNA expression.

## Simple Summary

More than half of patients with high-risk neuroblastoma (NB) relapse, likely driven by chemoresistant minimal residual disease in the bone marrow (BM-MRD). Although several quantitative PCR (qPCR) and droplet digital PCR (ddPCR) assays measuring different but overlapping sets of NB-associated mRNA (NB-mRNAs) have demonstrated a significant prognostic value at various time points, the optimal combination of MRD markers (a set of NB-mRNAs) and evaluation timing remain uncertain. Here, we evaluated BM-MRD with a 7NB-mRNAs ddPCR assay quantifying CRMP1, DBH, DDC, GAP43, ISL1, PHOX2B, and TH mRNAs across clinically relevant treatment stages. We analyzed 89 bone marrow samples obtained from mostly overlapping 30 high-risk NB patients at four time points: diagnosis (Dx), end of induction (EOI), end of high-dose chemotherapy (EOH), and end of consolidation (EOC). At EOH and EOC time points, BM-MRD was significantly associated with subsequent relapse. Moreover, patients with higher BM-MRD levels (7NB-mRNAs ≥ 3.5) had inferior 3-year event-free survival. These findings suggest that EOH and EOC are clinically informative evaluation time points for BM-MRD detected by 7NB-mRNAs expression.

## 1. Introduction

Neuroblastoma (NB) is the most common extracranial solid malignancy of childhood, accounting for approximately 8–10% of pediatric cancers and ~15% of pediatric cancer–related deaths [1]. In Japan, more than 150 children are newly diagnosed with NB each year [2]. NB arises from neural crest-derived cells of the sympathetic nervous system and exhibits marked clinical and biological heterogeneity, ranging from spontaneously regressing tumors in infants to aggressively progressive disease in older children [3,4]. Accordingly, newly diagnosed NB patients are stratified into low-, intermediate-, and high-risk groups based on established clinical and biological prognostic factors [5,6]. While outcomes are excellent for low- and intermediate-risk patients with >90% long-term survival, high-risk patients remain below 60% long-term survival [6,7].

High-risk NB is treated with intensified multimodal therapy consisting of induction therapy (chemotherapy and surgical resection), consolidation therapy (single or tandem high-dose chemotherapy with autologous peripheral blood stem cell transplantation (PBSCT) and radiation), and post-consolidation therapy (anti-GD2 immunotherapy and isotretinoin) [7,8]. Although nearly 80% of high-risk NB patients respond to induction therapy, many harbor minimal residual disease (MRD) that can later drive relapse. Consequently, more than half of patients with high-risk NB experience relapse, which remains the leading cause of cancer-related death in this population. Accurate MRD assessment is therefore critical for monitoring disease burden and treatment response and for improving risk-adapted management [9,10].

For three decades, MRD assessment in NB has relied on sets of NB-associated mRNAs (NB-mRNAs) to detect NB cells in bone marrow (BM), peripheral blood (PB), and peripheral blood stem cell (PBSC) samples, because NB lacks highly recurrent, universally applicable genomic aberrations [11,12]. Among the available approaches, quantitative PCR (qPCR) and droplet digital PCR (ddPCR) assays that quantify different but overlapping sets of NB-mRNAs have shown a significant prognostic value for MRD in BM (BM-MRD), PB (PB-MRD), and PBSC (PBSC-MRD) [13,14,15,16,17,18,19,20,21].

The clinical significance of MRD in NB depends on the sample type (BM, PB, or PBSC), the MRD markers (a set of NB-mRNAs), and the timing of evaluation [22]. Because BM is the most common site of relapse, BM-MRD has been studied most extensively. However, prior studies assessed BM-MRD at different time points and reported inconsistent results. At diagnosis (Dx), some studies found BM-MRD to be prognostic [14,16,19], whereas others reported no significant association with outcomes [18,23]. Likewise, BM-MRD after the end of consolidation (EOC) was associated with relapse in one study [18], but not at EOC in another study [17]. These discrepancies underscore the need to define clinically informative evaluation time points. Because the expression profiles of NB-mRNAs may vary at different evaluation time points, it is important to determine the optimal combination of MRD markers (a set of NB-mRNAs) and evaluation time points for BM-MRD evaluation.

In the present study, we evaluated BM-MRD with a 7NB-mRNA ddPCR assay quantifying CRMP1, DBH, DDC, GAP43, ISL1, PHOX2B, and TH mRNA at four time points: Dx, end of induction (EOI), end of high-dose chemotherapy (EOH), and EOC [18,24]. A total of 89 BM samples (25 Dx, 22 EOI, 22 EOH, and 20 EOC) were collected from mostly overlapping 30 high-risk NB patients and subjected to BM-MRD evaluation.

## 2. Materials and Methods

### 2.1. Patients and Samples

This study included 30 patients with high-risk neuroblastoma (NB) who provided bone marrow (BM) samples at one or more of the following time points: diagnosis (Dx), end of induction (EOI), end of high-dose chemotherapy (EOH), and/or end of consolidation (EOC) between December 2012 and November 2023. Patients were diagnosed and risk-stratified according to the Children’s Oncology Group (COG) Neuroblastoma Risk Stratification System [25,26] or the International Neuroblastoma Risk Group (INRG) Classification System [5]. All patients were treated at Kobe University Hospital, Kobe Children’s Hospital, or Tottori University Hospital using the standard high-risk NB regimen in Japan, based on the JN-H-07 [27], JN-H-11 [28], or JN-H-15 (UMIN000016848) protocol of the Japanese Children’s Cancer Group (JCCG) Neuroblastoma Committee (JNBSG). Treatment consisted of induction therapy (five cycles of induction chemotherapy with or without surgical resection) followed by consolidation therapy (single high-dose chemotherapy followed by autologous peripheral blood stem cell transplantation (PBSCT), with or without surgical resection, and radiotherapy). With written informed consent, BM samples were collected at the following time points: Dx (before initiation of treatment), EOI (after completion of induction therapy), EOH (after hematopoietic recovery following single high-dose chemotherapy and autologous PBSCT), and EOC (after completion of consolidation therapy). This study was conducted in accordance with the Clinical Research guidelines of Kobe University Graduate School of Medicine and was approved by the Institutional Review Boards of Kobe University Graduate School of Medicine (No. 180278), Kobe Children’s Hospital (No. 30-80), and Tottori University Hospital (No. 18A218).

### 2.2. 7NB-mRNAs ddPCR Assay

7NB-mRNAs ddPCR assay was performed as previously described [18]. Briefly, nucleated cells were isolated from BM samples using a Lymphoprep (Abbott Diagnostics Technologies, Oslo, Norway). Total RNA was extracted with a TRIzol Plus RNA Purification Kit (Life Technologies, Carlsbad, CA, USA). cDNA was synthesized from 0.5 to 1.0 µg of total RNA using a QuantiTect Reverse Transcription Kit (Qiagen, Valencia, CA, USA) and stored at −80 °C until analysis. Expression of 7 NB-mRNAs (CRMP1, DBH, DDC, GAP43, ISL1, PHOX2B, and TH mRNAs) and the reference gene mRNA (HPRT1 mRNA) was quantified on a QX200 ddPCR system (Bio-Rad Laboratories, Hercules, CA, USA) in accordance with the MIQE framework and the digital MIQE guidelines for reporting quantitative PCR and digital PCR experiments [29,30]. The combined 7NB-mRNA level (7NB-mRNAs) was defined as a weighted sum of the relative copy numbers for the seven targets; for each NB-mRNA, the weight was set as the reciprocal of the 90th percentile value measured in non-NB BM control samples, as previously reported [18].

### 2.3. Statistical Analysis

Differences in 7NB-mRNA levels between the two groups were assessed using the Mann–Whitney U test. Receiver operating characteristic (ROC) curves were constructed to evaluate the ability of 7NB-mRNA levels to discriminate patients with and without relapse, and the area under the curve (AUC) was calculated. AUC values of 0.50–0.69 were considered low accuracy, 0.70–0.89 moderate/significant accuracy, and 0.90–1.00 high accuracy [31]. Threshold value (TV) was determined as the point that maximized the Youden index (sensitivity + specificity − 1) on the ROC curve. The cut-off value was then chosen to optimize sensitivity and specificity, with a preference for specificity, and was subsequently rounded to a clinically meaningful value. Event-free survival (EFS) and overall survival (OS) were estimated using the Kaplan–Meier method and compared with the log-rank test. EFS was defined as the time from diagnosis to relapse or progression, and OS as the time from diagnosis to death. All tests were two-sided, and *p* < 0.05 was considered statistically significant. Statistical analyses were performed using EZR (version 1.68), a graphical user interface for R (version 4.23) based on R Commander (version 2.92) [32].

## 3. Results

### 3.1. Patient Characteristics and Sample Availability

A total of 30 high-risk NB patients were included (Table 1). They showed the typical characteristics of high-risk NB. Most patients were ≥18 months of age (86.7%, 26/30) and had primary adrenal tumors (80.0%, 24/30). BM metastasis at diagnosis was present in 90.0% (27/30). Histology was classified as NB in 73.3% (22/30), and tumors were undifferentiated or poorly differentiated in 76.7% (23/30); MYCN amplification was observed in 43.3% (13/30). All patients received the standard high-risk regimen in Japan, which included induction therapy (five cycles of induction chemotherapy with or without surgical resection) and consolidation therapy (single high-dose chemotherapy followed by autologous PBSCT, with or without surgical resection, and radiation), but not post-consolidation therapy (anti-GD2 immunotherapy and isotretinoin). During follow-up (median, 48.5 months; range, 16–121 months), 20 relapse events and 13 deaths occurred. The estimated 3-year EFS and OS were 50.0% (95% confidence interval (CI), 31.3–66.1%) and 66.2% (95% CI, 46.3–80.2%), respectively.

A total of 89 BM samples were obtained from 30 high-risk NB patients at four time points: Dx, 25 samples; EOI, 22 samples; EOH, 22 samples; and EOC, 20 samples. On average, 3.0 BM samples were collected per patient: 1 sample (*n* = 5), 2 samples (*n* = 4), 3 samples (*n* = 8), and 4 samples (*n* = 13).

### 3.2. BM-MRD Dynamics Across Dx, EOI, EOH, and EOC Time Points

**Figure 1:**
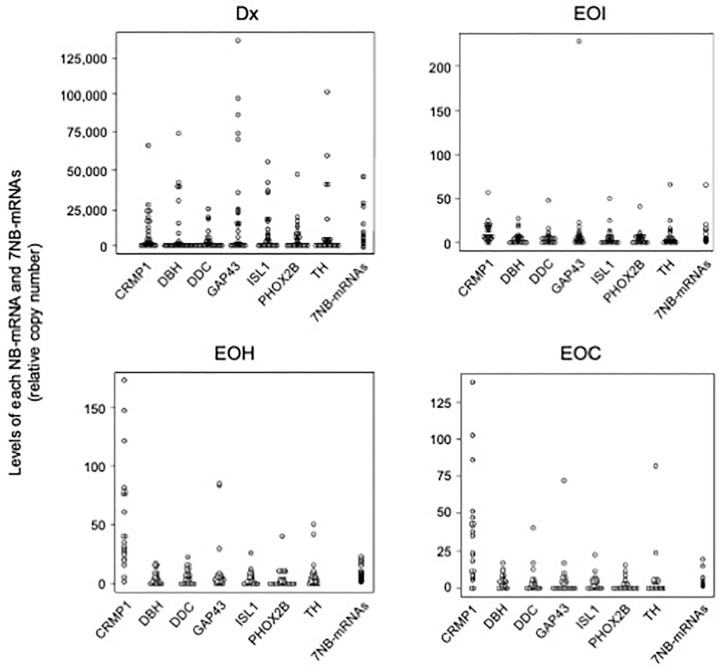
Levels of each NB-mRNA and 7NB-mRNAs in BM samples at Dx, EOI, EOH, and EOC time points of high-risk NB patients. The 89 BM samples at Dx, EOI, EOH, and EOC time points were collected from 25, 22, 22, and 20 high-risk NB patients, respectively, and were subjected to a 7NB-mRNA ddPCR assay. NB-mRNA, neuroblastoma-associated mRNA; Dx, diagnosis; EOI, end of induction; EOH, end of high-dose chemotherapy; EOC, end of consolidation; CRMP1, collapsin response mediator protein 1; DBH, dopamine beta-hydroxylase; DDC, dopa decarboxylase; GAP43, growth-associated protein 43; ISL1, ISL LIM homeobox 1; PHOX2B, paired-like homeobox 2b; TH, tyrosine hydroxylase.

To examine the clinical significance of BM-MRD at Dx, EOI, EOH, and EOC time points, we first determined the levels of each NB-mRNA (CRMP1, DBH, DDC, GAP43, ISL1, PHOX2B, and TH mRNA) and 7NB-mRNAs in a total of 89 (25 Dx, 22 EOI, 22 EOH, and 20 EOC) BM samples by ddPCR. As summarized in Figure 1, each NB-mRNA and 7NB-mRNAs were detected in 75.3% (469/623) and 100% (89/89) of analyzed samples, and their overall levels were gradually decreased during the course of treatment. As described before [18,33], 7NB-mRNAs were constantly detected in all BM samples at Dx, EOI, EOH, and EOC time points, whereas each NB-mRNA was variably detected in BM samples at different time points: Dx, 92% (161/175); EOI, 69.5% (107/154); EOH, 73.4% (113/154); and EOC, 62.9% (88/140). These results indicated the superior sensitivity of 7NB-mRNAs compared to each NB-mRNA and provided the rationale for the BM-MRD detected by 7NB-mRNA expression in the present study.

### 3.3. Association Between Relapse and BM-MRD at Dx, EOI, EOH, and EOC Time Points

**Figure 2:**
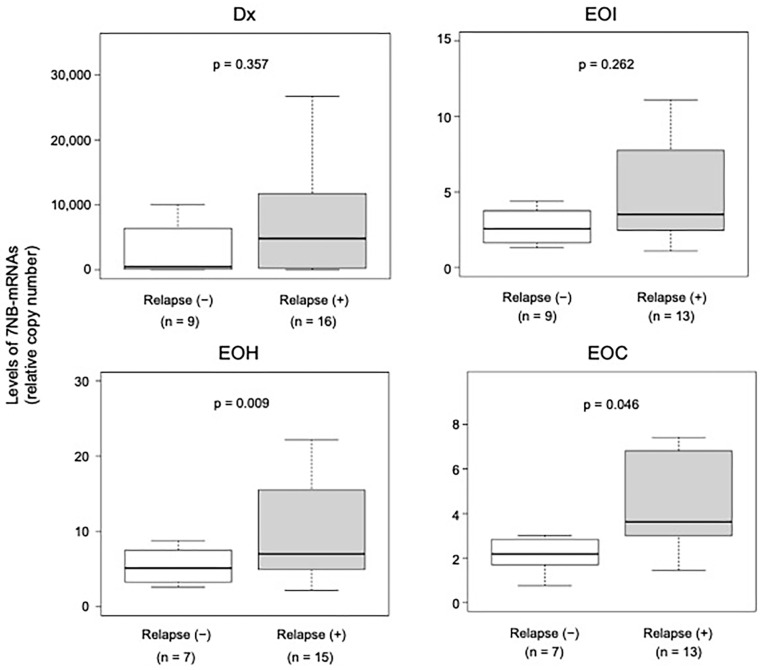
Association between relapse and BM-MRD at Dx, EOI, EOH, and EOC time points. Levels of 7NB-mRNAs were compared between BM samples without and with relapse at each time point. NB-mRNA, neuroblastoma-associated mRNA; Dx, diagnosis; EOI, end of induction; EOH, end of high-dose chemotherapy; EOC, end of consolidation.

We next compared the levels of BM-MRD detected by 7NB-mRNAs expression between patients without and with relapse at each time point (Figure 2). BM-MRD levels tended to be higher in patients with relapse at all time points. Differences were not statistically significant at Dx or EOI time points. However, BM-MRD levels were significantly higher in patients with relapse at EOH (*p* = 0.009) and EOC (*p* = 0.046) time points.

**Figure 3:**
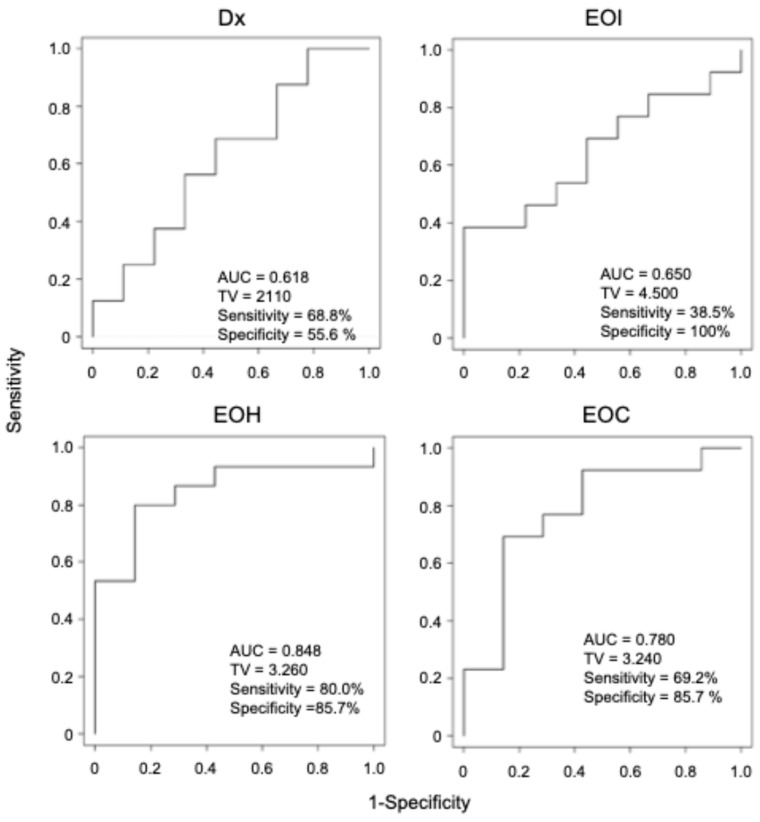
ROC analyses of BM-MRD at Dx, EOI, EOH, and EOC time points. ROC curves were plotted for levels of 7NB-mRNAs in BM samples without and with relapse at each time point. Dx, diagnosis; EOI, end of induction; EOH, end of high-dose chemotherapy; EOC, end of consolidation; AUC, area under curve; TV, threshold value.

To evaluate the ability to discriminate patients with and without relapse, we then generated receiver operating characteristic (ROC) curves for BM-MRD at each time point and calculated area under the curve (AUC) values (Figure 3). AUC values were 0.618 at Dx and 0.650 at EOI (both < 0.70; low accuracy), 0.848 at EOH, and 0.780 at EOC (both ≥ 0.7; significant accuracy). Together, these results indicated that BM-MRD at EOH and EOC time points were significantly associated with relapse.

### 3.4. Survival Stratification by BM-MRD at EOH and EOC Time Points

**Figure 4:**
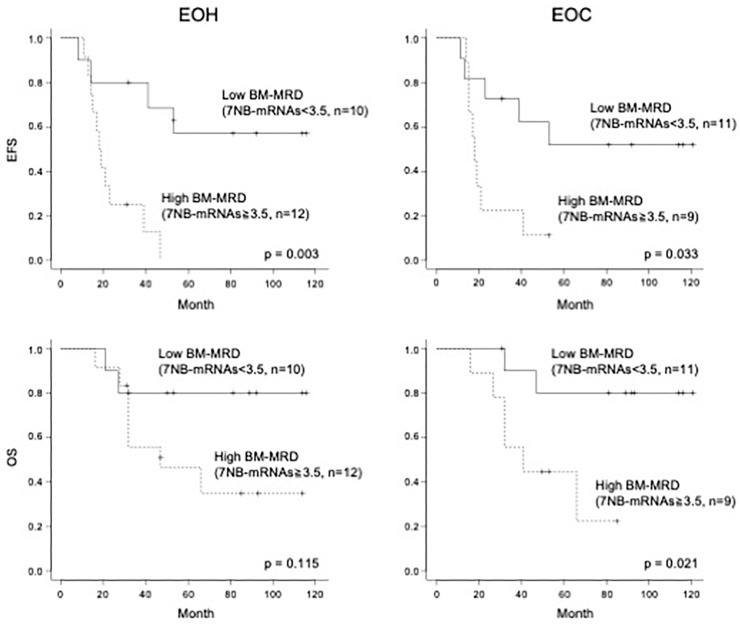
Kaplan–Meier analyses for high-risk NB patients dichotomized by BM-MRD at EOH and EOC time points. Kaplan–Meier curves were plotted for EFS and OS of high-risk NB patients with high and low BM-MRD at EOH and EOC time points. BM-MRD, minimal residual disease in bone marrow; NB-mRNA, neuroblastoma-associated mRNA; EOH, end of high-dose chemotherapy; EOC, end of consolidation; EFS, event-free survival; OS, overall survival; dotted line, high BM-MRD; solid line, low BM-MRD.

Given the significant association at EOH and EOC time points, we dichotomized patients at those time points into high and low BM-MRD groups and performed Kaplan–Meier analyses (Figure 4). Based on ROC analyses, cut-off values were selected to optimize sensitivity and specificity, with a preference for specificity, and then rounded to a clinically meaningful threshold of 3.5 at both EOH and EOC time points.

At the EOH time point (*n* = 22), the estimated 3-year EFS and OS were 50.0% (95% CI, 28.2–68.4%) and 66.0% (95% CI, 41.5–82.2%), respectively. Patients with high BM-MRD (7NB-mRNAs ≥ 3.5; *n* = 12) had a significantly lower 3-year EFS than those with low BM-MRD (7NB-mRNAs < 3.5; *n* = 10): 25.0% (95% CI, 6.0–50.5%) versus 80.0% (95% CI, 40.9–94.6%) (*p* = 0.003). Three-year OS was 55.6% (95% CI, 23.7–78.7%) in the high BM-MRD group and 80.0% (95% CI, 40.9–94.6%) in the low BM-MRD group (*p* = 0.115).

At the EOC time point (*n* = 20), the estimated 3-year EFS and OS were 50.0% (95% CI, 27.1–69.2%) and 73.9% (95% CI, 48.2–88.2%), respectively. The high BM-MRD group (7NB-mRNAs ≥ 3.5; *n* = 9) was associated with significantly worse outcomes than the low BM-MRD group (7NB-mRNAs < 3.5; *n* = 11): 3-year EFS, 22.2% (95% CI, 3.4–51.3%) versus 72.7% (95% CI, 37.1–90.3%) (*p* = 0.033); and 3-year OS, 55.6% (95% CI, 20.4–80.5%) versus 90.0% (95% CI, 47.3–98.5%) (*p* = 0.021). These results demonstrated that BM-MRD at EOH and EOC time points had a significant impact on clinical outcomes.

## 4. Discussion

In the present study, we evaluated BM-MRD with a 7NB-mRNA ddPCR assay in 89 BM samples collected at 4 time points (Dx, EOI, EOH, and EOC) from mostly overlapping 30 high-risk NB patients. BM-MRD at EOH and EOC, but not Dx and EOI, was significantly associated with relapse risk and survival outcomes (Figure 2, Figure 3 and Figure 4). To our knowledge, this is the first report demonstrating a significant prognostic value of BM-MRD at the EOH time point in high-risk NB patients.

At the EOH time point, BM sampling was performed just after hematopoietic recovery following single high-dose chemotherapy and autologous PBSCT. The observed prognostic impact suggests that relapse-causing NB cells can persist in the BM compartment at this early post-transplantation stage. Such cells may originate from the recipient (survived NB cells during high-dose chemotherapy) and/or the donor (survived/contaminated NB cells in autologous PBSC graft). As exemplified by the clinical observation that BM is the most common site of relapse in high-risk NB patients, NB cells may have therapy resistance and growth advantage and become the relapse-causing NB cells in the BM microenvironment composed of multiple stromal cell types, even after myeloablative high-dose chemotherapy. Although the clinical significance of BM-MRD at EOH has not been reported, several studies have shown that PBSC-MRD is associated with patient outcomes in high-risk NB patients [20,21,34]. Taken together, EOH may represent a particularly informative time point for BM-MRD evaluation in future studies.

At the EOC time point, BM samples were collected after completion of single high-dose chemotherapy followed by autologous PBSCT, with or without surgical resection and radiation [27,28]. In contrast to a previous study [17], we observed a significant association between BM-MRD at EOC and patient outcomes (Figure 2, Figure 3 and Figure 4).

In the present study, BM-MRD at Dx did not show prognostic significance (Figure 2 and Figure 3), although several studies have reported an association between BM-MRD at Dx and patient outcomes [14,19,35]. Likewise, BM-MRD at EOI was not significantly associated with patient outcomes, contrary to multiple prior reports [14,16,19,23,36]. These discrepancies may arise from differences in patient cohort composition, treatment protocols, and MRD markers (a set of NB-mRNAs).

The present data demonstrated that BM-MRD detected by 7NB-mRNAs’ expression had a significant predictive power of relapse in high-risk NB patients, with EOH and EOC emerging as the most informative time points. Clinically, these time points may be particularly useful to stratify high-risk NB patients for the optimal post-consolidation therapies, enabling us to incorporate the BM-MRD evaluation into a high-risk NB treatment protocol.

However, this study has limitations. One limitation is the number of patients and samples. The small number of patients and samples at each time point (*n* = 20–25) reduced the statistical power, increasing the likelihood of false-negative findings and restricting the generalizability of our results beyond the present cohort. The other limitation is the treatment heterogeneity, especially in post-consolidation therapy. All patients received the standard high-risk NB regimen in Japan, in which post-consolidation therapy was not uniformly administered as frontline treatment and was applied variably as second-line therapy.

## 5. Conclusions

In summary, we analyzed the clinical significance of BM-MRD at Dx, EOI, EOH, and EOC time points and revealed that EOH and EOC are prognostic evaluation time points for BM-MRD detected by 7NB-mRNAs expression. Notably, an earlier EOH time point provides better timing for BM-MRD evaluation in future studies.

## Figures and Tables

**Table 1:** Patient and sample characteristics.

Variable	Category	Count
Patient		
Age at diagnosis	<18 months	4 patients (13.3%)
	≥18 months	26 patients (86.7%)
Sex	Male	22 patients (73.3%)
	Female	8 patients (26.7%)
Primary tumor site	Adrenal gland	24 patients (80.0%)
	Mediastinum	1 patient (3.3%)
	Retroperitoneum	2 patients (6.7%)
	Neck mass	2 patients (6.7%)
BM metastasis at diagnosis	(−)	3 patients (10.0%)
	(+)	27 patients (90.0%)
Histology	NB	22 patients (73.3%)
	GNB nodular	5 patients (16.7%)
	GNB intermixed	1 patient (3.3%)
	GN maturing	0 patient (0.0%)
Differentiation grade	Undifferentiated	3 patients (10.0%)
	Poorly differentiated	20 patients (66.7%)
	Differentiating	0 patient (0.0%)
Ploidy	Diploid	17 patients (56.7%)
	Hyperdiploid	4 patients (13.3%)
	Diploid and Hyperdiploid	2 patients (6.7%)
MYCN status	Non-amplified	14 patients (46.7%)
	Amplified	13 patients (43.3%)
Observation period	Median	48.5 months
	Range	16–121 months
Relapse	(−)	10 patients (33.3%)
	(+)	20 patients (66.7%)
Survival	3-year EFS	50.0% (95% CI: 31.3–66.1%)
	3-year OS	66.2% (95% CI: 46.3–80.2%)
Sample		
Collection time point	Dx	25 patients (83.3%)
	EOI	22 patients (73.3%)
	EOH	22 patients (73.3%)
	EOC	20 patients (66.7%)
Collected sample per patient	1 sample/patient	5 patients (16.7%)
	2 samples/patient	4 patients (13.3%)
	3 samples/patient	8 patients (26.7%)
	4 samples/patient	13 patients (43.3%)

BM, bone marrow; NB, neuroblastoma; GNB, ganglioneuroblastoma; GN, ganglioneuroma; MYCN, MYCN proto-oncogene bHLH transcription factor; EFS, event-free survival; OS, overall survival; Dx, diagnosis; EOI, end of induction; EOH, end of high-dose chemotherapy; EOC, end of consolidation.

## Data Availability

The data generated and analyzed in this study are available upon reasonable request.

## Abbreviations

The following abbreviations are used in this manuscript:

NB	Neuroblastoma
MRD	Minimal residual disease
NB-mRNAs	Neuroblastoma-associated mRNAs
BM	Bone marrow
BM-MRD	MRD in BM
PB	Peripheral blood
PB-MRD	MRD in PB
PBSC	Peripheral blood stem cell
PBSCT	PBSC transplantation
PBSC-MRD	MRD in PBSC
qPCR	Quantitative PCR
ddPCR	Droplet digital PCR
Dx	Diagnosis
EOI	End of induction
EOH	End of high-dose chemotherapy
EOC	End of consolidation
COG	Children’s Oncology Group
INRG	International Neuroblastoma Risk Group
JCCG	Japanese Children’s Cancer Group
JNBSG	JCCG Neuroblastoma Committee
MIQE	Minimum Information for Publication of Quantitative Digital PCR Experiments
ROC	Receiver operator characteristic
AUC	Area under curve
EFS	Event-free survival
OS	Overall survival
CI	Confidence interval
